# Analysis of microarrays of miR-34a and its identification of prospective target gene signature in hepatocellular carcinoma

**DOI:** 10.1186/s12885-017-3941-x

**Published:** 2018-01-03

**Authors:** Fang-Hui Ren, Hong Yang, Rong-quan He, Jing-ning Lu, Xing-gu Lin, Hai-Wei Liang, Yi-Wu Dang, Zhen-Bo Feng, Gang Chen, Dian-Zhong Luo

**Affiliations:** 1 Department of Pathology, First Affiliated Hospital of Guangxi Medical University, 6 Shuangyong Road, Nanning, Guangxi Zhuang Autonomous Region 530021 People’s Republic of China; 2 Department of Ultrasonography, First Affiliated Hospital of Guangxi Medical University, 6 Shuangyong Road, Nanning, Guangxi Zhuang Autonomous Region 530021 People’s Republic of China; 3 Center for Genomic and Personalized Medicine, Guangxi Medical University, 22 Shuangyong Road, Nanning, Guangxi Zhuang Autonomous Region 530021 People’s Republic of China; 4 Department of Hepatobiliary Surgery, First Affiliated Hospital of Guangxi Medical University, 6 Shuangyong Road, Nanning, Guangxi Zhuang Autonomous Region 530021 People’s Republic of China

**Keywords:** Hepatocellular carcinoma, miRNA-34a, Gene expression omnibus, Gene ontology, Network analysis

## Abstract

**Background:**

Currently, some studies have demonstrated that miR-34a could serve as a suppressor of several cancers including hepatocellular carcinoma (HCC). Previously, we discovered that miR-34a was downregulated in HCC and involved in the tumorigenesis and progression of HCC; however, the mechanism remains unclear. The purpose of this study was to estimate the expression of miR-34a in HCC by applying the microarray profiles and analyzing the predicted targets of miR-34a and their related biological pathways of HCC.

**Methods:**

Gene expression omnibus (GEO) datasets were conducted to identify the difference of miR-34a expression between HCC and corresponding normal tissues and to explore its relationship with HCC clinicopathologic features. The natural language processing (NLP), gene ontology (GO), pathway and network analyses were performed to analyze the genes associated with the carcinogenesis and progression of HCC and the targets of miR-34a predicted in silico. In addition, the integrative analysis was performed to explore the targets of miR-34a which were also relevant to HCC.

**Results:**

The analysis of GEO datasets demonstrated that miR-34a was downregulated in HCC tissues, and no heterogeneity was observed (Std. Mean Difference(SMD) = 0.63, 95% confidence intervals(95%CI):[0.38, 0.88], *P* < 0.00001; P_heterogeneity_ = 0.08 I^2^ = 41%). However, no association was found between the expression value of miR-34a and any clinicopathologic characteristics. In the NLP analysis of HCC, we obtained 25 significant HCC-associated signaling pathways. Besides, we explored 1000 miR-34a-related genes and 5 significant signaling pathways in which CCND1 and Bcl-2 served as necessary hub genes. In the integrative analysis, we found 61 hub genes and 5 significant pathways, including cell cycle, cytokine-cytokine receptor interaction, notching pathway, p53 pathway and focal adhesion, which proposed the relevant functions of miR-34a in HCC.

**Conclusion:**

Our results may lead researchers to understand the molecular mechanism of miR-34a in the diagnosis, prognosis and therapy of HCC. Therefore, the interaction between miR-34a and its targets may promise better prediction and treatment for HCC. And the experiments in vivo *and vitro* will be conducted by our group to identify the specific mechanism of miR-34a in the progress and deterioration of HCC.

**Electronic supplementary material:**

The online version of this article (10.1186/s12885-017-3941-x) contains supplementary material, which is available to authorized users.

## Background

Liver cancer ranks as the second leading cause of cancer death in men in less developed countries and sixth in more developed countries [1]. During 2012, 745,500 deaths occurred out of estimated 782,500 new HCC cases worldwide, with nearly 50% happening in China [1].High prevalence is observed in parts of East and South-East Asia where chronic hepatitis B virus (HBV) and hepatitis C virus (HCV) infection are epidemic [2]. Hepatocellular carcinoma (HCC) is recognized as the most common type of liver cancer, accounting for 90% of primary liver cancer. There exist difficulties in treating HCC due to a series of sequential and complex processes involved in the carcinogenesis and progression of HCC. Although radiation, surgery, liver transplantation or chemotherapy are widely used in the therapy of HCC, the survival rate of HCC patients is still less than 5% [3]. Therefore, it is urgent to enhance our knowledge of molecular pathogenesis of HCC and explore novel biomarkers in favor of therapy of HCC. Gene signatures would provide efficient molecular basis of the clinicopathological features for characterizing the heterogeneity of HCC. In addition, the regulatory pathways and networks involved in the mechanism of HCC would lead to the identification of molecular fingerprints for directing therapeutic strategies.

MicroRNAs(miRNAs) are composed of a family of endogenous, noncoding small RNA molecules(∼18–25 nucleotides long), which serve as post-transcriptional gene expression regulators via binding to the 3′--untranslated regions(3’-UTRs) region of their target messenger RNAs(mRNA) [4]. Besides, miRNAs participated in multiple biological processes in oncology via inhibiting translation of mRNA or degrading mRNA [5–8]. Several studies have identified that ectopic expression of miR-34a is related to cell cycle, proliferation, migration, invasion,apoptosis and prognosis of cancers by targeting AXL/ SIRT1/Yin Yang-1 [9–12]. Several studies have shown that miR-34a regulates the carcinogenesis and progression of cancers via modulation of Notch1 Pathway, SIRT1/p53 pathway, WNT/TCF7 signaling [13–15]. Kang et al. and Zhou et al. have reported that miR-34a is associated with chemo-resistance [13, 16]. One study conducted by Cao et al. has found that miR-34a could influence the impact of lincRNA-UFC1 on proliferation and apoptosis in HCC cells [17]. In our previous study, we demonstrated that miR-34a decreased in HCC, and in vitro experiment has also identified that miR-34a could inhibit cell proliferation, invasion and migration, and increase caspase activity and cellular apoptosis by modulating phospho-ERK1/2 and phospho-stat5 signaling, as well as the level of c-MET [18]. However, the molecular mechanism of miR-34a in HCC tumorigenesis and development remains unclear. Therefore, a systemic and comprehensive understanding of miR-34a-target genes and relevant signaling pathways is important for diagnosis, therapy and prognosis of HCC.

In this study, GEO datasets were applied to verify the associations between miR-34a expression and HCC. Additionally, we performed a series of analyses to assess the miR-34a-predicted genes which are associated with carcinogenesis, progression and chemo-resistance in HCC. We then evaluated the potential value of miR-34a in HCC diagnosis, prognosis and therapy with network and pathway analyses.

## Methods

### Comprehensive analysis of miR-34a expression in HCC based on GEO datasets

The expression data of miR-34a was collected from the GEO(http://www.ncbi.nlm.nih.gov/geo/) databases up to January 2016 with the following keywords: (“HCC” OR “hepatocellular carcinoma” OR “liver cancer” OR “liver carcinoma” OR “liver malignan*” OR “liver neoplasm”) and (“miRNA” OR “microRNA”). Inclusion criteria were as follows: (1) both HCC tissues and adjacent HCC tissues (or healthy liver tissues) or only HCC tissues were included in each dataset with each group containing more than two samples; (2) the dataset sample organism was *Homo sapiens*; (3) the expression data of miR-34a (has-miR-34a or has-miR-34a-5p) from the experimental and control groups could be acquired or calculated. Expression values of miR-34a and sample size in both test and control groups were calculated. Moreover, means and standard deviations of these values were extracted to estimate the different levels of miR-34a in case and control groups by Review Manager (Revman Version 5.3, Copenhagen, Denmark) with random-effects model. The chi-square test and the I2 statistic were applied to evaluate the heterogeneity across studies. Heterogeneity was considered to exist when the *P* value < 0.05 or I^2^ > 50%. Further, SMD and its 95%CI were pooled to evaluate the stability of the analysis. It was considered to be statistically significant if the corresponding 95%CI for the pooled SMD did not overlap 1 or −1. Additionally, for sensitivity analysis, we eliminated every study to evaluate the source of heterogeneity.

### NLP analysis of HCC

#### Extraction and filtering of data

**Fig. 1:**
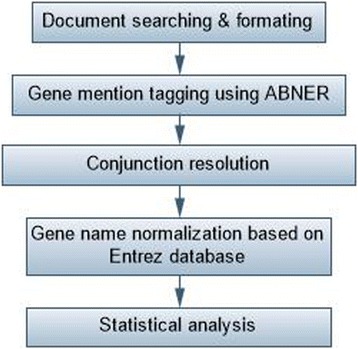
Flow chart of the natural language processing (NLP) analysis of hepatocellular carcinoma

We searched PubMed in an attempt to identify all relevant studies published between January 1980 and May 2015. Publications were retrieved with the following key words: (hepatocellular carcinoma) and (resistance or prognosis or metastasis or recurrence or survival or carcinogenesis or sorafenib or bevacizumab) and (“1980/01/01” [PDAT]: “2015/05/25” [PDAT]). All genes and proteins related to the key words were extracted and gathered in a list with the following of gene mention tagging by applying A Biomedical Named Entity Recognizer (ABNER, an open source tool for automatically tagging genes, proteins and other entity names in text, http://pages.cs.wisc.edu/bsettles/abner/) [19] and conjunction resolution. The flow chart of NLP analysis was shown in Fig. 1.

#### Statistical analysis

The frequency was calculated for each gene occurrence in the NLP analysis. The higher frequency implies the stronger association between HCC and certain genes. The number of all eligible literatures in PubMed database was recorded as N. The frequency of certain gene and HCC in PubMed was marked as “m” and “n”, respectively. It was denoted as “k” when a gene and HCC occurred simultaneously. Then we calculated the possibility of frequency greater than “k” co-citation via hypergeometric distribution in completely random conditions.

$$ \mathrm{p}=1-\sum \limits_{\mathrm{i}=0}^{\mathrm{k}-1}\mathrm{p}\left(i\left|\mathrm{n},\kern0.5em \mathrm{m},\kern0.5em \mathrm{N}\right.\right) $$

$$ \mathrm{p}\left(i\left|\mathrm{n},\kern0.5em \mathrm{m},\kern0.5em \mathrm{N}\right.\right)=\frac{n!\left(N-n\right)!m!\left(N-m\right)}{\left(n-i\right)!i!\left(n-m\right)!\left(N-n-m+i\right)!N!} $$

#### The comprehensive analysis of HCC- related genes

We conducted GO analysis on the functions of differently expressed genes in HCC and classified the related genes into three major groups: biological processes, cellular component and molecular functions. Pathway analysis was applied in GenMAPP v2.1 for Kyoto Encyclopedia of Genes and Genomes (KEGG) pathway enrichment and the value of P was calculated to select the significantly enriched pathway. We also combined three different interactions: 1) protein interaction, gene regulation, protein; 2) modification of the existing high-throughput experiments; 3) interactions between genes mentioned previously. In addition, the pathway data was downloaded from KEGG database and the interactions between genes were analyzed by KEGGSOA (http://www.bioconductor.org/packages/2.4/bioc/html/ KEGGSOAP.html) package from R (http://www.r-project.org/), including enzyme–enzyme relation, protein–protein interaction and gene expression interaction [20].

We downloaded the data of protein interactions from MIPS database (http://mips.helmholtz-muenchen.de/proj/ppi/) [21]. For the interaction mentioned previously, algorithm co-citation in the abstracts in PubMed was used to analyze gene term and all term variants co-occurring with the certain gene. And we also calculated the frequency of the cocitation genes. Then we conducted statistical analyses according to the description in NLP analysis. Finally, medusa software was used to perform the network.

#### Prediction of miRNA-34a target genes

We analyzed the predicted targets of miR-34a by applying 11 independent software: DIANA-microT, MicroInspector, miRanda, MirTarget2, miTarget, NBmiRTar, PicTar, PITA, RNA22, RNAhybrid and TargetScan. The results were considered reliable when the results were calculated by four or more software. GO analysis, pathway analysis and network analysis of miR-34a target genes were performed in accordance with the same principles of genes from NLP analysis.

#### Integrative analysis of miR-34a targets and the natural language processing

We calculated the overlap of miR-34a targets predicted in silico and HCC related-genes acquired from NLP analysis. And the ingenuity pathway analysis was carried out to show the relationships between miR-34a and its target genes associated with HCC. The overlap of the miR-34a target genes predicted by in-silico bioinformatics tools and HCC-related genes was performed subsequently.

## Results

### Comprehensive analysis of GEO datasets

**Fig. 2:**
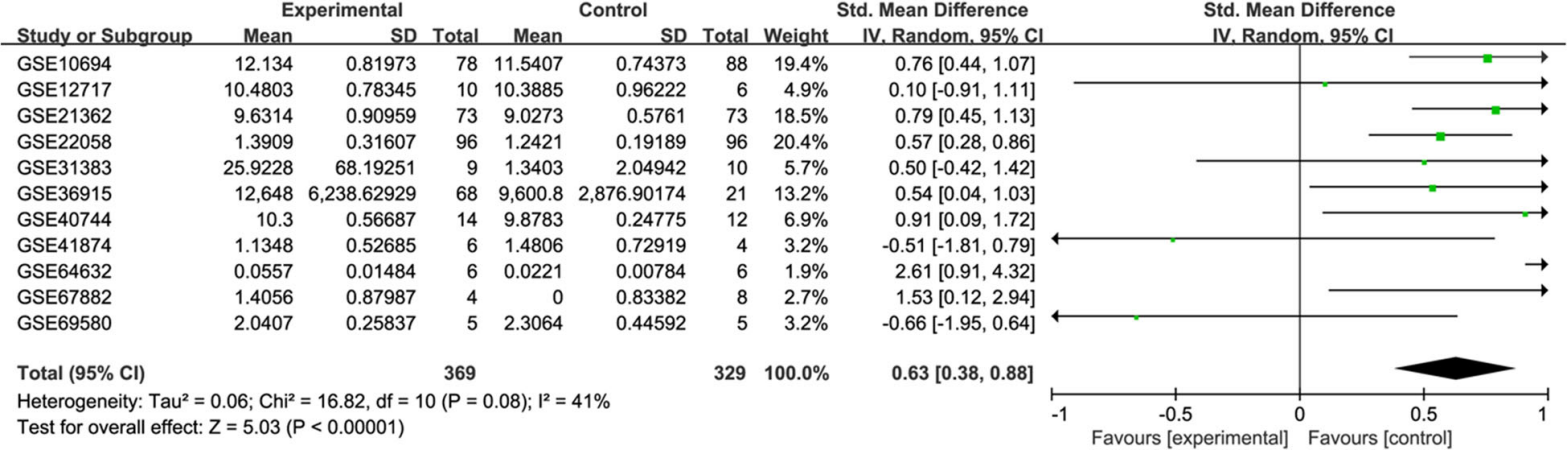
Forest plot showing SMD of miR-34a expression between HCC tissues and corresponding normal tissues

**Fig. 3:**
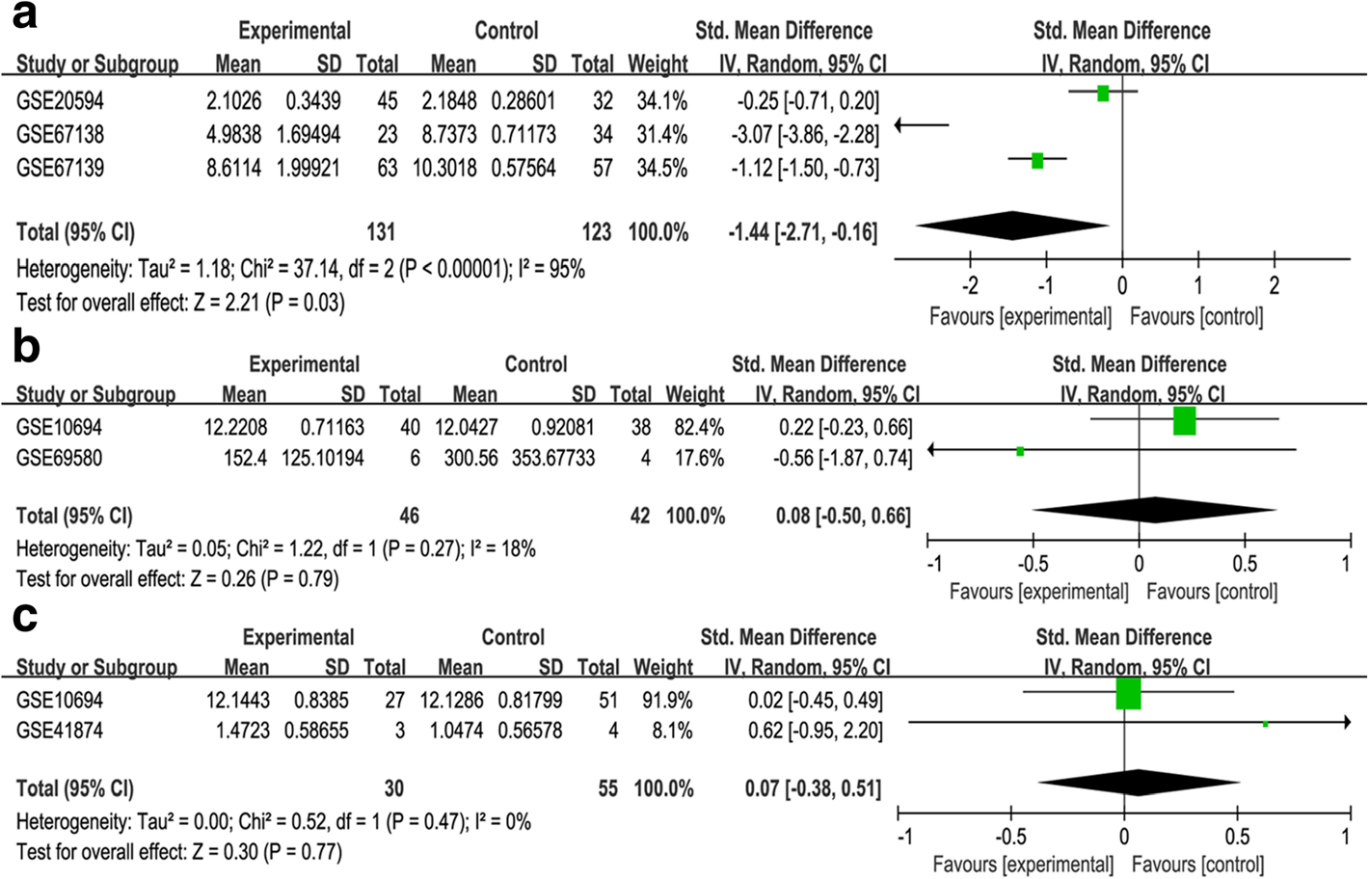
Forest plot showing the association between miR-34a expression and HCC clinocopathologic characteristics. **a** Forest plot showing SMD of miR-34a expression in HCC tissues with or without vascular invasion. **b** Forest plot showing SMD of miR-34a expression in HCC tissues with or without cirrhosis. **c** Forest plot showing SMD of miR-34a expression in HCC tissues with or without metastasis

Fourteen eligible datasets were involved in this study, which included 3 datasets containing HCC tissues and 11 datasets containing both HCC and adjacent tumor (or healthy) tissues (Table 1). The result showed that the expression level of miR-34a in HCC tissues was statistically lower than that in normal tissues with no heterogeneity (SMD = 0.63, 95%CI [0.38, 0.88], *P* < 0.00001; P _heterogeneity_ = 0.08 I^2^ = 41% Figure 2).The three studies which contained HCC tissues only were used to evaluate the difference of miR-34a expression in HCC patients with or without vascular invasion. The comparison identified that miR-34a expression was not associated with vascular invasion in HCC patients (SMD = −1.44, 95%CI: [−0.16,-2.71], *P* = 0.03; P_heterogeneity_ < 0.00001 I^2^ = 95%, Figure 3a).Besides, there existed significant heterogeneity after we had reviewed every study. Among the 11 datasets mentioned above, two datasets (GSE69580, GSE10694) were applied to estimate the relationship of miR-34a with cirrhosis in HCC patients. Another two datasets (GSE10694, GSE41874) were performed to analyze whether the expression of miR-34a was related to metastasis in patients with HCC. Finally, no relationship was observed between miR-34a expression and cirrhosis or metastasis (P_cirrhosis_ = 0.79, Fig. 3b; P_metastasis_ = 0.77, Figure 3c).

**Table 1:** Characteristics of miR-34a gene expression used in the analysis of GEO datasets

GEO accession	Author	Sample size		Country	Platform
		HCC patients	Healthy controls		
GSE10694	Gu et al.	78	88	China	GPL6542
GSE12717	Su et al.	10	6	China	GPL7274
GSE22058	Burchard	73	73	USA	GPL10457
GSE21362	Sato et al.	96	96	Japan	GPL10312
GSE31383	Hoshida et al.	9	10	USA	GPL10122
GSE36915	Lee et al.	68	21	Taiwan	GPL8179
GSE40744	Farci et al.	14	12	USA	GPL14613
GSE41874	Okano et al.	6	4	Japan	GPL7722
GSE64632	Selaru et al.	6	6	USA	GPL18116
GSE67882	Banerjee et al.	4	8	India	GPL10850
GSE69580	Hung et al.	5	5	Taiwan	GPL10850
GSE20594	Hoshida et al.	45	32	USA	GPL10122
GSE67138	Chuang et al.	23	34	USA	GPL8786
GSE67139	Chuang et al.	63	57	USA	GPL8786

### NLP analysis of HCC

#### The analysis of HCC-related genes

A total of 64,577 articles for HCC-related molecules were identified for initial browse after primary search in PubMed. A total of 1800 HCC-relevant genes were obtained. All of these cancer-related genes were categorized into different biological processes, cellular components, molecular functions according to GO analysis (Additional file 1: Table S1). In the pathway analysis, we found 25 signaling pathways were significant (*P* < 0.005), which has been discussed in our previously published work [20]. By constructing gene network, we found the hub genes functioned as a key factor in regulating the stability of the network. In our previous study, the gene network of those 1800 genes were performed [20].The published article also showed that several hub genes were highly related to other genes: PIK3CA, PIK3R2, MAPK1, MAPk3, JAK2, EGFR, KRAS, NRAS [20].

#### The analysis of miR-34a predicted targets

In the present study, we analyzed the potential targets of miR-34a to explore the biological function of miR-34a relying on its target-protein-coding genes. One thousand potential genes were identified and categorized in GO analysis (Additional file 2: Table S2). Subsequent to the pathway analysis,five pathways were discovered to be statistically significant: Focal adhesion, p53 signaling pathway, Cell cycle, Cytokine-cytokine receptor interaction, Notch signaling pathway (*P* < 0.05, Table 2).

**Table 2:** Pathway analysis of miR-34a-related genes

term	count	P value	genes
hsa04510:Focal adhesion	8	5.09E-04	CCND1, MAP2K1, PGF, BCL2, MET, VEGFA, PDGFRA, RELN
hsa04115:p53 signaling pathway	5	0.001385856	CCNE2, CCND1, SERPINE1, CDK6, IGFBP3
hsa04110:Cell cycle	6	0.001921952	CCNE2, E2F3, CCND1, E2F5, CDK6, CDC25A
hsa04060:Cytokine-cytokine receptor interaction	6	0.039495189	CCL22, MET, VEGFA, PDGFRA, KITLG, KIT
hsa04330:Notch signaling pathway	3	0.045372273	NOTCH2, NOTCH1, JAG1

The genes were obtained from the natural language processing (NLP) analysis and 5 signaling pathways were significant (*P* < = 0.05)

**Fig. 4:**
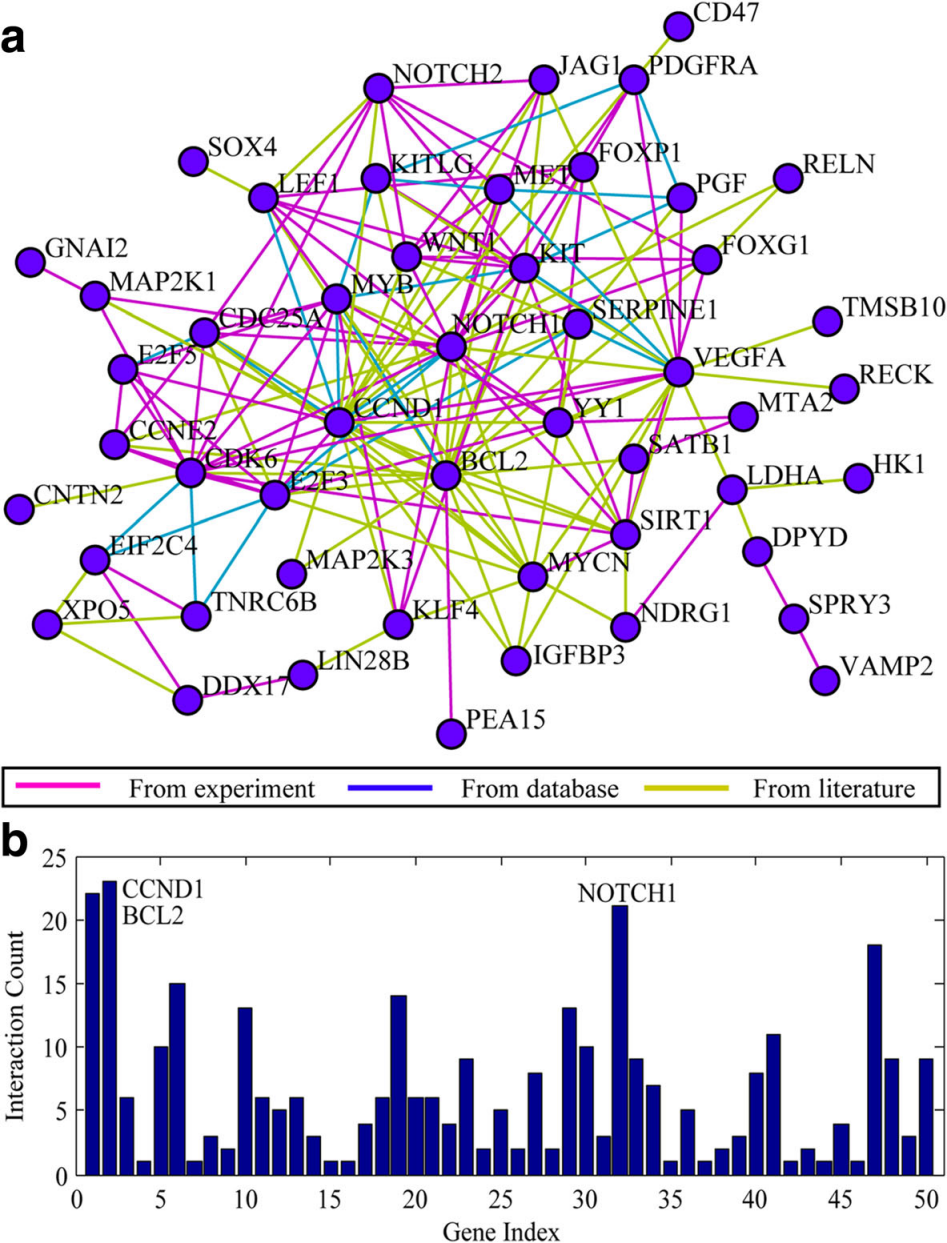
Network analysis and connectivity analysis of miR-34a targets. **a** Network analysis of miR-34a targets. Brown represents association, green represents inhibition and blue represents activation. **b** Connectivity analysis of miR-34a targets. The connectivity of Bcl-2 is the highest one which has a total of twenty-two related-genes (z-test, *P* = 0.0037)

In the network analysis, only two genes are statistically significant and both of them were the highest hub genes, including CCND1 and BCL2 (Fig. 4c, Figure 4d). CCND1 participates in the regulation of the three pathways mentioned in Table 2 (focal adhesion, p53 signaling pathway, cell cycle) and BCL-2 acts as a member of focal adhesion. In addition, the expression of CCND1 and BCL-2 were both associated with the biological processes described in GO analysis: cell cycle and proliferation, stress response, developmental processes, protein metabolism.

#### Integrative analysis of miR-34a target genes and the NLP results

**Fig. 5:**
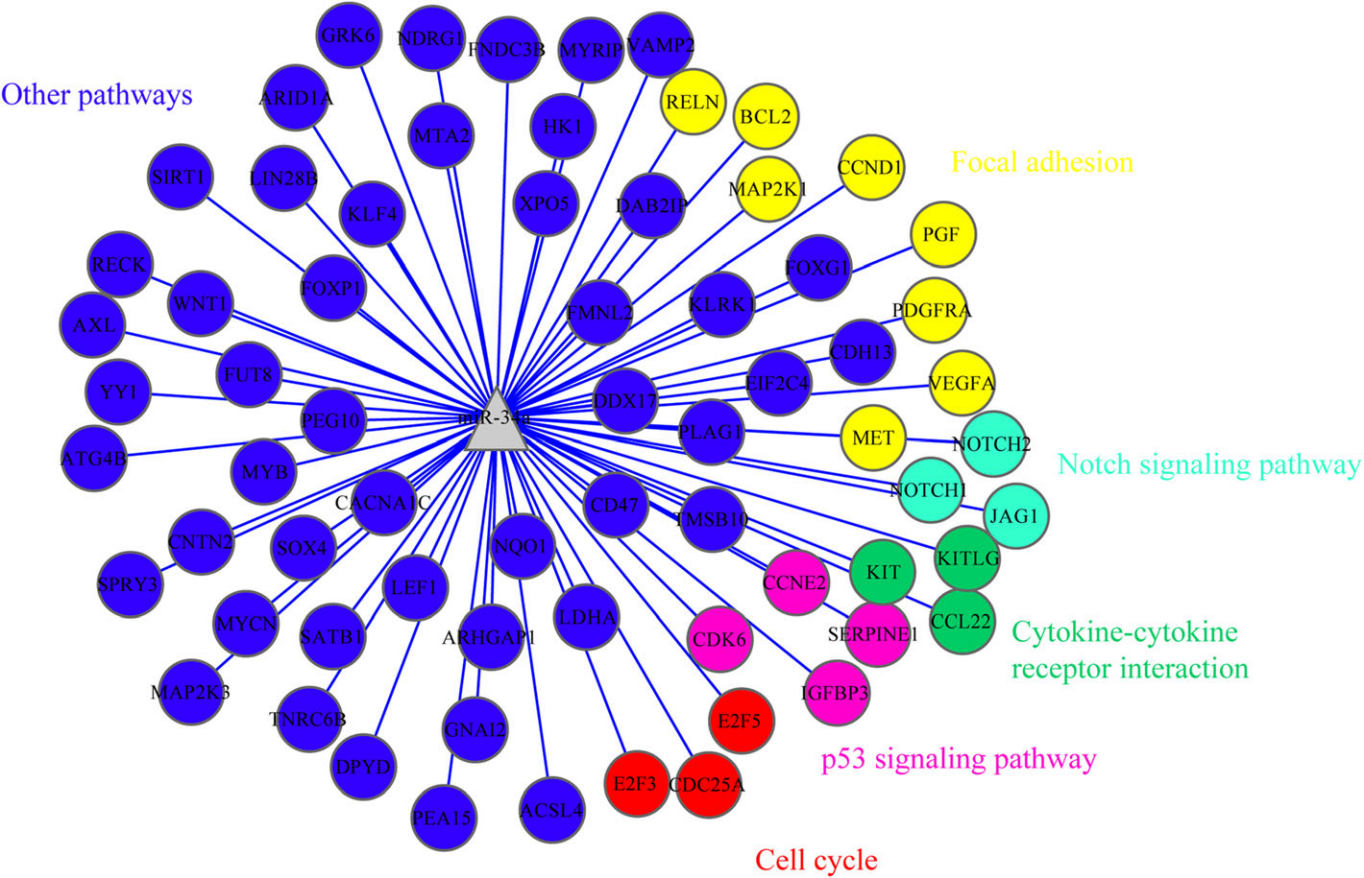
Integrative -analysis of miR-34a target genes and the NLP results. Sixty-nine overlapping genes and their functional pathway that are not only associated with the molecular mechanism of HCC but also are the potential miR-34a target genes were obtained in this final integrative-analysis

In the integrative analysis, we calculated the overlap of miR-34a targets and HCC-related genes obtained from the NLP analysis. As a result, 61 HCC-related genes, such as VEGFA, Bcl-2, CCND1, MET, KIT, Notch1, DPYD, SERPINE1 and CDK6 were potentially modulated by miR-34a, were summarized in Additional file 3: Table S3. Besides, the relationship among these genes was shown in Fig. 5, including five significant pathways: cell cycle (*P* = 0.001921952); cytokine-cytokine receptor interaction (*P* = 0.039495189); notching signaling pathway (*P* = 0.045372273); p53 pathway (*P* = 0.001385856) and focal adhesion (*P* = 5.09E-04). Among the five pathways mentioned above, the three genes (E2F3, E2F5, CDC25A) were involved in the cell cycle pathway, and KIT/CCL22 were found in the cytokine-cytokine receptor interaction pathway. Besides, Notch1/Notch2/JAG1 and CDK6/IGFPB3/CCNE2 were significantly related to the notching signaling pathway and p53 pathway, respectively. Additionally, we concluded that VEGFA, Bcl-2, CCND1, MET, PDGFRA were related to the functions of focal adhesion in HCC patients.

## Discussion

Nowadays, in order to prolong the survival time of HCC patients, more and more therapies are applied in the clinical stage, including radiotherapy, interventional operation, combined therapy. However, the survival rate is still unsatisfactory. In recent years, more and more researches have focused on the molecular targeted therapy and made exciting progress. Liu et al. have concluded that miR-222 and miR-494 could enhance HCC patients’ resistance to sorafenib by activating the PI3K/AKT signaling pathway [22, 23]. The study reported by Lin et al. has also identified that miR-21 partially reduced the cytotoxic effects of sorafenib in combination with matrine against HCC [24]. However, another two reports have verified that increased expression of miRNAs could assist the efficiency of sorafenib treatment for HCC patients [25, 26]. In our previous study, we identified that increased expression of miR-34a might help the diagnosis and prognosis of HCC by regulating c-MET [18]. Although numerous articles have verified the functions of miR-34a in the carcinogenesis and progression of HCC, the molecular mechanism of miR-34a related to HCC still remains unclear. Therefore, this article was the first to identify the relationship of miR-34a expression with HCC based on microarray data. In addition, we separately analyzed the related pathways of HCC-relate genes and miR-34a targets, and further explored the potential molecular mechanism of miR-34a by overlapping genes associated with HCC and miR-34a.

The consequences of GEO analysis testified that miR-34a expression was downregulated in HCC tissues, which was consistent with our previous study [18]. On the contrary, another two articles have demonstrated that upregulation of miR-34a could promote the proliferation of HCC [27, 28]. The research conducted by Gougelet et al. has identified that the higher expression of miR-34a induced by activation of β-catenin could enhance the risk of HCC by targeting CCND1 [27]. However, the other study has testified that Aflatoxin-B1 (AFB1) might contribute to the progression of HCC by upregulating miR-34a, which may down-regulate Wnt/β-catenin signaling pathway [28]. Besides, due to the limitation of sample size, no statistical significance was found in the associations of miR-34a expression value with HCC clinicopathological features. Nevertheless, some other studies have shown that the low expression of miR-34a might promote the progression of HCC.

Two researchers have suggested that ectopic expression of miR-34a was related to tumor metastasis and invasion via modulating c-MET signaling pathway [18, 29]. Cheng et al. have also provided evidence that abrogation functions of miR-34a could contribute to HCC development through cell cycle pathway and p53 pathway [30]. Moreover, it has been demonstrated that miR-34a/ toll-like receptor 4(TLR4) axis may function as a key regulator in increasing the risk of HCC [31]. Besides, another article has verified that overexpression of miR-34a could improve the effect of cisplatin treatment by enhancing cytotoxicity of NK cells for HCC patients [32]. In addition, Lou et al. have shown that decreased expression of miR-34a may reduce the sensitivity of HCC cells to quercetin by upregulating SIRT1 and downregulating p53 [11]. Therefore, the functions of miR-34a in HCC are complex and still needed further investigation.

In order to explore the molecular mechanism of miR-34a in moderating the progression of HCC, we conducted a series of bioinformatics analyses. The results showed that among the targets of miR-34a, the most significant hub genes were CCND1 and Bcl-2, which were parts of focal adhesion, p53 signaling pathway, cell cycle pathway. Meanwhile, we also found these two genes (CCND1 and Bcl-2) were included in the hub genes which were calculated from the interactive analysis of miR-34a and the NLP analysis. MiR-195 could suppress the development of cancers by targeting CCND1 [33]. Besbes et al. have also concluded that decreased expression of Bcl-2 family members contributed to the progression of apoptosis in cancers [34]. Especially, Gougelet et al. and Zhu et al. have suggested that the feedback loop composed of miR-34a/β-catenin/CCND1 played a critical role in regulating the progression of HCC [27, 28]. Hence, miR-34a may serve as an important regulator in the development of HCC by targeting CCND1 and Bcl-2.

In the interactive analysis, we not only calculated the hub genes, but also the statistically significant pathways involved in moderating the progression of miR-34a-related HCC. The outcome showed eight genes-VEGFA, Bcl-2, CCND1, MET, NOTCH1, SERPINE1, DYPD and CDK6-had the highest connectivity. It was worth noticing that the eight genes were classified into five significantly different pathways. It suggested that miR-34a may impact the development and progression of HCC by moderating cell cycle, cytokine-cytokine receptor interaction, notching pathway, p53 pathway and focal adhesion. Thus, the functions of every genes involved in the five pathways were essential in understanding the effects of miR-34a on HCC patients.

### Cell cycle

E2F3 and E2F5 are members of the E2F family which is composed of transcription factors and attributed to the cell cycle progression by regulating G1/S-phase transition [35]. Some studies have shown that miR-503 and miR-195 could inhibit the G1/S transition by suppressing the expression of E2F5 [36, 37]. And the downregulation of E2F5 has been identified to be associated with development of HBV-related HCC [38]. Several articles have suggested that miRNAs could suppress the proliferation, metastasis and invasion of HCC cells by targeting E2F3 [39–41]. Additionally, it has been verified that the inhibition of E2F3 induced by overexpression of miRNAs could enhance the sensitivity of HCC patients to anti-cancer agents and decelerate the progression of HCC [39, 42].

### Cytokine-cytokine receptor interaction

C-kit is the receptor of stem cell factor (SCF), and the activation of c-kit have been suggested to be crucial for cell proliferation and migration [43]. The activation of c-kit has been suggested to be attributed to the cell proliferation and cirrhosis of HCC [43, 44]. Yang et al. have proved that the activation of TGF-β-miR-34a-CCL22 signaling could promote the progression of portal vein tumor thrombus in HCC patients [45]. Two studies have also proved that miRNAs could promote the development of HCC by blocking G1/S transition via reducing expression of CDK6 [36, 46].

### Notching pathway

Notching signaling pathway composed of notch receptors(Notch1–4) and notch ligands(Jag1) is critical for determining cell fates and associated with therapy of HCC [47, 48]. The result reported by Xue et al. indicated that JAG1/Notch1 signaling is positively associated with the extrahepatic metastasis in HCC by moderating the level of osteopontin (OPN) [49]. However, Wang et al. have concluded that increased expression of Notch1/Jagged1 could promote the progression of HCC via inhibiting beta-catenin expression [50]. Another two studies have indicated that Notch1 may be a therapeutic target by downregulating Wnt/β-catenin pathway and CyclinD1/CDK4 pathway in HBV-associated HCC [51, 52]. In addition, it has been demonstrated that inhibition of Notch2 regulated by C8orf4 could suppress the self-renewal of liver cancer stem cells(CScs) and Notch2 and Jag1 may function as novel therapeutic targets for HCC treatment [53, 54].

### P53 pathway

P53 is widely recognized as a tumour suppressor in regulating cell cycle, apoptosis, metabolism and DNA repair [55]. Giacoia et al. have concluded that wide-type p53 could upregulate the expression of miR-107 and then reduce the level of CDK6 and Notch2, which suppresses glioma cell growth [56]. It has been indicated that overexpression of plasminogen activator inhibitor-1 (PAI-1/SERPINE1) could enhance tumour cell proliferation as well as inhibit G(1)-phase transition complexes, cdk4/6/ cyclin D3 and promote the cell-cycle suppressors p53, p27Kip1 and p21Cip1/Waf1 [57]. IGFBP3 induced by p53 have also been verified to be related to the apoptosis of HCC cells [58].

### Focal adhesion

The pathway analysis also suggests that Bcl-2, CCND1, PDGFRA, VEGFA and c-MET belong to the focal adhesion pathway. It has also been suggested that CCND1 variants may be positively correlated with the precancerous cirrhosis of hepatocarcinogenesis [59]. Increased expression of platelet-derived growth factor receptor alpha (PDGFRA) promoted by miR-146a has been verified to be associated with the microvascular invasion and poor prognosis of HCC [60]. In addition, it has been shown that the synergism of sorafenib has contributed to the therapy of HCC patients by suppressing the level of MET [61]. It has been identified that HCC patients with amplification of vascular endothelial growth factor A (VEGFA) are more likely to be sensitive to sorafenib [62]. Zhou et al. have also found that miR-503 serve as a suppressor of tumor angiogenesis by targeting VEGFA in HCC patients [63]. However, the functions of genes which were related with miR-34a expression and progression of HCC have not been identified.

In the present study, researchers have paid attention to the results of the comprehensive analyses, especially the section of the integrative –network analysis of miR-34a targets and HCC-related genes. It illustrated the most important genes and pathways involved in the functions of miR-34a in HCC, which may offer researchers more insights into understanding the relationship between miR-34a and HCC. However, the targets of miR-34a were predicted in silico, including genes verified or not verified in experiments, which could make it difficult to explore the exact mechanism of miR-34a in HCC. It is noted that the two genes CCND1 and bcl-2 seem to serve as key regulators between the expression of miR-34a and HCC. Therefore, it is imperative to find out the exact relationships among miR-34a, CCND1/bcl-2 and HCC at a further step.

## Conclusions

In summary, it was worth considering that the analyses of miR-34a- targets in HCC would provide effective guidelines for the diagnosis, prognosis and therapy of patients with HCC. The results indicate that miR-34a primarily controls cell cycle, cytokine-cytokine receptor interaction, notching pathway, p53 pathway and focal adhesion pathway in regulating the tumorigenesis and process of HCC. Therefore, these pathways may offer novel insights into the functions of miR-34a in HCC and guide the researchers to find more effective methods to the prevention and treatment of HCC.

## Additional files

Additional file1: Table S1. GO enrichment analysis of HCC related genes. (XLSX 12 kb)

Additional file2: Table S2. GO enrichment analysis of miR-34a target genes. (XLSX 14 kb)

Additional file3: Table S3. Integrative-analysis of miR-34a target genes and the natural language processing (NLP) results. Twenty-four overlapping genes that are not only associated with the development and progression of HCC but also are the potential miR-34a target genes were obtained in this integrative–analysis (XLSX 14 kb)

## Abbreviations

3’-UTRs: 3′--untranslated regions
95%CI: 95% confidence intervals
AFB1: Aflatoxin-B1
GEO: Gene expression omnibus
GO: Gene ontology (GO)
HBV: Hepatitis B virus
HCC: Hepatocellular carcinoma
HCV: Hepatitis C virus
KEGG: Kyoto Encyclopedia of Genes and Genomes
miRNA: microRNA
mRNA: Messenger RNAs
NLP: Natural language processing
PDGFRA: Platelet-derived growth factor receptor alpha
SMD: Std. mean difference
TLR4: Toll-like receptor 4
VEGFA: Vascular endothelial growth factor A
